# Flaxseed protein content prediction based on hyperspectral wavelength selection with fractional order ant colony optimization

**DOI:** 10.3389/fnut.2025.1551029

**Published:** 2025-04-15

**Authors:** Bo Wang, Junying Han, Chengzhong Liu, Jianping Zhang, Yanni Qi

**Affiliations:** ^1^College of Information Science and Technology, Gansu Agricultural University, Lanzhou, China; ^2^Crop Research Institute, Gansu Academy of Agricultural Sciences, Lanzhou, China

**Keywords:** hyperspectral imaging, wavelength selection, visible-near infrared, protein content, fractional order ant colony optimization

## Abstract

The protein content of flaxseed (*Linum usitatissimum*) is a crucial factor influencing its nutritional value and quality. Spectral technology combined with advanced modeling methods offers a fast, accurate, and cost-effective approach for predicting protein content. In this study, visible-near infrared hyperspectral imaging (VNIR-HIS) technology was combined with fractional order ant colony optimization (FOACO) to determine the protein content of flaxseed. Thirty flaxseed varieties commonly cultivated in Northwest China were selected, and hyperspectral data along with protein content measurements were collected. A joint x-y distance algorithm was applied to divide the dataset into calibration and prediction sets after removing outliers. Partial least squares regression (PLSR) models were developed based on both raw and preprocessed spectra, with the Savitzky-Golay (SG) smoothing method found to provide superior performance. The performance of wavelength selection methods based on FOACO, principal component analysis (PCA), and ant colony optimization (ACO) was compared using PLSR and multiple linear regression (MLR) models. The FOACO-MLR model achieved a prediction accuracy of 0.9248, a root mean square error (RMSE) of 0.4346, a relative prediction deviation (RPD) of 3.6458, and a mean absolute error (MAE) of 0.3259. The results show that the FOACO-MLR model provides significant advantages in predicting flaxseed protein content, particularly in terms of prediction accuracy and stability of characteristic bands. By combining VNIR-HIS technology with the FOACO wavelength selection algorithm, this study offers an efficient and rapid method for determining the protein content of flaxseed, providing reliable technical support for the precise detection of nutritional components.

## 1 Introduction

Whole flaxseed comprises 30−41% fat, 20–35% dietary fiber, 20–30% protein, 4–8% water, 3–4% ash, and 1% simple sugars (1). Flaxseed protein is particularly rich in essential amino acids, notably lysine and arginine, which contribute significantly to cardiovascular health and immune function (2). Beyond being a vital nutrient component, protein also plays a crucial role in determining the flavor and texture of flaxseed products. The accurate measurement of protein content is essential not only for assessing its nutritional and health benefits but also for influencing the market positioning of the product. With advancements in technology, the continuous innovation in modern testing methods provides more efficient and precise approaches to meet the increasing demand for food quality and safety in the marketplace.

Currently, the protein content in flaxseed is primarily assessed through chemical and instrumental analysis methods (3). Chemical analysis methods include the traditional Kelectroschner method (4) and the Dumas combustion method (5), both of which estimate protein content indirectly by measuring the nitrogen content of the sample. While these methods are accurate, they often require extended time and complex operational procedures (6). In contrast, instrumental analysis methods, such as visible and near-infrared hyperspectral imaging system (VNIR-HIS) (7) and fourier transform infrared spectroscopy (FTIR) (8), enable rapid determination of protein content by analyzing the absorption properties of a sample at specific wavelengths. These techniques are non-destructive, rapid, and efficient, making them particularly suitable for large-scale detection and quality control. The principle behind VNIR-HIS technology involves capturing the reflection and absorption information of the sample at various wavelengths to provide detailed spectral data for each pixel (9). Each pixel contains a set of spectral data that reflects the material composition at that specific location. Consequently, the spectrum provides compositional information about the sample. The integration of spectral analysis with stoichiometry enables the detection of the chemical composition of the sample (10, 11). As a non-destructive testing method, spectroscopy technology allows for rapid analysis of sample composition while obtaining high-precision compositional information without altering the physical structure of the seed. Thus, VNIR-HIS technology can detect not only the protein content of flax but also analyze the distribution, shape, and other characteristics of flaxseeds. This approach facilitates the combination of component detection and spatial distribution analysis, thereby offering comprehensive data support for food processing and quality control.

In addition to providing useful spectral data, complete hyperspectral images may contain numerous uncorrelated variables, which can diminish the robustness and prediction accuracy of the calibration model (12). Each sample point typically encompasses hundreds or even thousands of bands of information, resulting in increased data redundancy. This redundancy not only escalates the burden of storage and computation but may also obscure critical feature information (12). Therefore, to mitigate redundancy while retaining the essential physical information of the spectra (13), band selection techniques have emerged to enhance analysis efficiency and model accuracy. Beyond traditional principal component analysis (PCA) (14), a variety of modern techniques are widely employed for band selection, including heuristic search-based algorithms such as genetic algorithm (GA) (15), particle swarm optimization (PSO) (16), and ant colony optimization (ACO) (17) etc.

Fractional calculus refers to the extension of traditional integer order calculus, wherein the order of differentiation can be any real or complex number. This mathematical framework is widely utilized across various fields, including mathematics, physics, engineering, control theory, and biology. Compared to traditional calculus, fractional calculus offers greater flexibility and expressiveness in addressing complex phenomena, particularly those involving memory effects, long-term memory, and nonlocality (18). In recent years, the application of fractional calculus to group intelligence has emerged as a promising area of research, garnering increasing attention. Researchers have begun to investigate its application to various types of differential equations, particularly in the context of fractional order evolutionary equations, optimal control, and optimal feedback control (19). Notably, the ACO has been enhanced through fractional calculus to modify the pheromone update mechanism (20). Furthermore, the combination of fractional order ant colony optimization (FOACO) with genetic algorithms has yielded improved results (21). The FOACO enhances the traditional pheromone updating mechanism by incorporating concepts from fractional calculus, resulting in a smoother and more flexible search process that is better suited for high-dimensional and complex optimization problems.

PCA is one of the most widely used unsupervised dimensionality reduction techniques (22). In the context of feature wavelength screening, PCA evaluates the significance of each wavelength by calculating its contribution to each principal component. Typically, the wavelength associated with the principal component that exhibits a higher contribution is regarded as the most representative feature wavelength. By retaining a selection of principal components, the dimensionality of the data can be reduced while preserving the most representative information (23). Although PCA is an effective method for dimensionality reduction in hyperspectral data processing, it has limitations, including the neglect of low variance features and assumptions of linearity.

The ACO is inspired by the foraging behavior of ants in nature (24). ACO enhances problem-solving by simulating the process of ants searching for food, wherein they gradually accumulate pheromones and select the shortest path. This algorithm possesses strong global search capabilities and effectively avoids becoming trapped in local optimal solutions (25). Consequently, ACO is extensively employed in hyperspectral band selection to identify the most informative subset among a vast array of bands, thereby improving both the predictive performance and stability of the model (20). ACO's advantage lies in its ability to guide the search process through the pheromone update mechanism, allowing the ants to progressively converge toward the global optimal solution within the solution space. Furthermore, ACO does not necessitate extensive prior knowledge and exhibits good flexibility and scalability. However, due to the localized nature of the pheromone updating process, the search may be less efficient and risk converging to a local optimal solution.

To address these challenges, the FOACO is introduced for hyperspectral band selection. This approach effectively mitigates the limitations of PCA and ACO in band selection by enhancing global search capabilities, smoothing path selection, and addressing nonlinear relationships. FOACO regulates the nonlinear pheromone diffusion process through the introduction of a smoothing mechanism based on fractional-order calculus, resulting in smoother pheromone updates and reducing instability associated with local update rules (18). This mechanism enhances the continuity and flexibility of path updating, improves global search capabilities, and prevents the traditional ACO from becoming trapped in local optimal solutions. Additionally, it addresses the shortcomings of PCA, which may overlook key features with low variance during band selection. Consequently, FOACO overcomes the limitations of both PCA and ACO in hyperspectral data analysis, significantly enhancing prediction accuracy and model stability, particularly in waveband selection and global search capabilities, thereby demonstrating clear advantages.

In this study, FOACO will be utilized to select the optimal bands to enhance the prediction accuracy of the protein content in flaxseed. By integrating the partial least squares regression (PLSR) model with the multiple linear regression (MLR) model, we will investigate the selection of the most informative bands from hyperspectral data and develop an accurate prediction model for protein content. Through the optimization of band selection, we aim to improve prediction accuracy and offer new insights and methodologies for the application of hyperspectral imaging technology in agricultural research.

## 2 Materials and methods

### 2.1 Experimental data set

The dataset in this study comprises 30 flaxseed varieties widely cultivated in northwestern China, as shown in Table 1. All varieties were harvested from the experimental bases of the flax breeding team at the Crop Institute, Gansu Academy of Agricultural Sciences, including the Zhangye Experimental Station in Gansu (100.37° E, 38.84° N), the Jingtai Experimental Station in Gansu (104.07° E, 37.18° N), the Lanzhou New Area Experimental Station in Gansu (103.70° E, 36.56° N), and the internal experimental station of the Academy (103.68° E, 36.09° N). After harvesting from the experimental fields, 20 plants were randomly sampled from each experimental plot. Following seed threshing, drying, and cleaning to remove chaff and residual seeds smaller than half the normal seed size, random sampling was conducted with four replicates. In each replicate, 30 g of seeds (with a moisture content of 9%) was weighed using an electronic balance with a precision of 1/1,000 g, placed in nylon mesh bags, and taken back to the laboratory. The samples were then left in a room-temperature, ventilated environment for 7 days before hyperspectral images were acquired. Once the hyperspectral images were obtained, the samples were immediately sent to the Gansu Academy of Agricultural Sciences for analysis of each variety's protein content, oil content, linoleic acid, and lignan.

**Table 1:** Flaxseed varieties.

**No**.	**Variety**
1	Onyc
2	Shuang You Ma 1
3	Shuang Ya 12
4	Shuang Ya 14
5	Shuang Ya 15
6	Zhang Ya 3
7	Ba 6
8	Ba 5
9	Ba 4
10	Ba 3
11	Hua Ya 5
12	Hua Ya 6
13	Ding Ya 17
14	Hei Ya 2
15	Ning Ya 10
16	Ba 9
17	Ba 11
18	Gan Ya 3
19	Yan Za 10
20	Jin Ya 7
21	Yi Ya 3
22	Ba Ya 18
23	Ba 14
24	901 Ba Ya 15
25	139 Ba Ya 17
26	Hua Ya l
27	Hua Ya 2
28	Hua Ya 3
29	Hua Ya 4
30	Ba 2

### 2.2 Hyperspectral bands—data collection and processing

#### 2.2.1 Hyperspectral imaging systems

**Figure 1:**
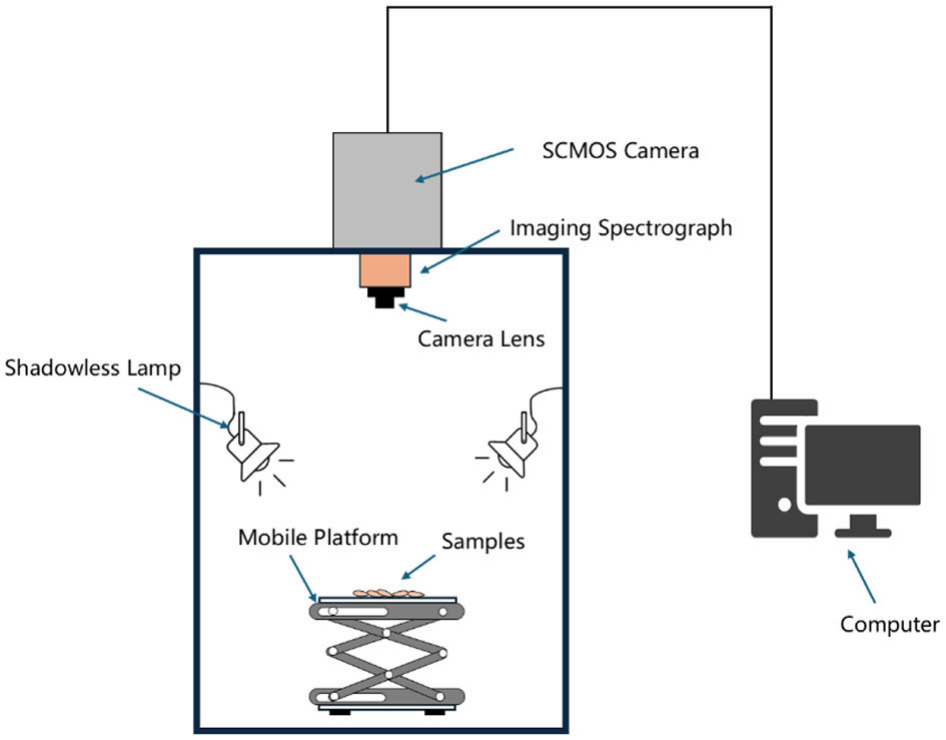
Hyperspectral imaging system.

The Gaia Field portable hyperspectral system, provided by Sichuan Shuangli Spectral Imaging Technology Co., LTD., is depicted in Figure 1. This system comprises the GaIAField-V10E hyperspectral camera, a 2,048 × 2,048 pixel imaging lens, an HSI-CT-150 × 150 standard white board (PTFE), an HISA-DB indoor imaging camera, four groups of shadow light sources, a HISA-TP-L-A tripod control, and hyperspectral data acquisition software known as Spec View. It features a spectral range of 380–1,018 nm, encompassing 320 bands, and offers a spectral resolution of 2.8 nm. The system has a numerical aperture of F/2.4, a slit size of 30 × 14.2 mm, utilizes SCMOS detectors, and supports a built-in push-scan and autofocus imaging mode with a 14-bit dynamic range. The core components of the system include a standardized light source, a spectral camera, an electronic control mobile platform, a computer, and control software. Its working principle involves push-scan imaging technology, which, in conjunction with the array detector and spectrometer, allows for real-time data collection as the slit and lens of the spectrometer move relative to each other, ultimately resulting in the assembly of a complete data cube.

#### 2.2.2 Image acquisition and calibration

Before image acquisition, the hyperspectrometer and darkbox light source were started and preheated for 30 min. The instrument parameters were configured, the camera exposure time was 49 ms, the gain was set to 2, the frame rate was 18.0018 Hz, and the forward scanning speed was 0.00643 cm/s. Among the 30 flaxseed varieties, 50 seeds were randomly selected as samples for hyperspectral image acquisition and placed in the darkbox on the mobile platform in sequence. Then these 50 seeds were used as the same region of interest (ROI) to obtain the average spectral curve of these 50 seeds. Each variety was collected three times, and finally all varieties were collected 90 times, a total of 4,500 seeds were scanned, and 90 average spectral curves were obtained. Due to the differences in the internal nutrients of individual seeds, the spectral curve may be biased. To reduce this effect, this study averaged the spectral curves of 50 seeds of each variety to establish a prediction model. This method helps to smooth small changes and reduce measurement noise, thereby providing a reliable representation of the spectral characteristics of each variety. The three repeated acquisitions of each variety further ensured the reliability of the data, enhanced the robustness of the data set, and facilitated subsequent analysis.

After acquisition, the original hyperspectral image was subjected to black and white correction to eliminate the dark current noise introduced by the camera. The correction formula is shown in Equation 1:


(1) $$\begin{array}{l} \begin{array}{c} R_{T}=\frac{I-I_{d}}{I_{w}-I_{d}} \end{array} \end{array}$$

After the original hyperspectral image was corrected for black and white to remove dark current noise, ENVI5.3 was used to define regions of interest (ROI) for flaxseeds and background. Then a support vector machine (SVM) was applied for supervised classification to distinguish between seeds and background. The classification results were converted into a vector to generate a mask image, which was used to remove background pixels. Finally, the average spectrum of all seed pixels in the mask region was calculated as the spectrum of the sample.

#### 2.2.3 Sample set segmentation

Several dataset delineation methods have been proposed, including Kennard-Stone (KS), Sample Set Delineation Based on Joint X-Y Distance (SPXY), and duplex (26). The SPXY algorithm extends the KS algorithm by incorporating spectral variables (x) and chemical values (y), thereby creating a more representative dataset (27). In this study, sample set partitioning based on the joint X-Y distance (SPXY) was used to allocate caraway seed protein content into modeling and prediction sets in a 2:1 ratio. The reasonableness of the sample division was assessed by calculating the maximum, minimum, mean, and standard deviation of the samples. The dataset consists of 90 samples, divided into a training set of 60 samples and a test set of 30 samples. The results are presented in Table 2, which illustrates the similarity of the mean values of protein content in both the test and training sets, confirming a consistent distribution across the groups. Therefore, the overall division of the sample set is deemed reasonable.

**Table 2:** Descriptive statistics for the training and test sets.

**Dataset**	**Protein**				
	**Number of samples**	**Maximum (%)**	**Minimum (%)**	**Average (%)**	**Standard deviation**
Training set	60	28.46	23.01	25.1	1.54
Testing set	30	27.76	23.07	25.21	1.28

#### 2.2.4 Spectral data pre-processing

Experimentally collected spectral information, while containing valuable data relevant to the sample, often includes interfering elements such as random noise, background interference, stray light, and spectral variations induced by the sampling device (28). Therefore, the application of spectral pre-processing is essential for removing irrelevant information and noise, which helps mitigate the effects of interference from scattering and background baseline drift in the spectral data prior to the establishment of wavelength selection methods. Spectral preprocessing enhances the model's capacity to account for spectral variations associated with compound concentrations, thereby improving both accuracy and predictive power. In this study, five distinct preprocessing methods were investigated. The first-order derivative (1D) technique reduces translational signals and eliminates interference caused by light scattering and path length variations (29). The Standard Normal Transform (SNV) removes bias and scale effects from the spectra, standardizing the spectral values at each wavelength point to achieve zero mean and unit variance, thus enhancing the comparability and accuracy of the data (30). Multiplicative scattering correction (MSC) enhances the accuracy and reliability of spectral data by correcting for scattering effects in the spectral signal and eliminating the influences of sample morphology and surface state (31). The moving average (MA) preprocessing method employs a sliding window to smooth the spectral data, reducing noise and minimizing frequency variations, which in turn improves signal stability and consistency (32). Lastly, the SG smoothing filter is a widely used technique for smoothing spectral data by fitting local polynomials, which reduces noise while preserving the morphology of spectral features (33).

### 2.3 Construction of wavelength selection method

#### 2.3.1 PCA

In the process of band selection, the spectral data of the training set are first standardized to ensure a consistent scale across each band, thereby mitigating the excessive influence of certain bands on the analysis results due to their large value ranges (34). PCA is then applied to reduce the dimensionality of the standardized data, selecting the top five principal components with the highest cumulative contribution rates to ensure that they explain the majority of the variability in the data. These principal components typically encapsulate the main information of the dataset while effectively eliminating redundancy and noise. By extracting the load values (eigenvectors) of each principal component, the contribution of each band to the principal component can be analyzed. The absolute value of the load indicates the importance of each band within the principal component, with larger load values signifying a stronger correlation with the principal component (23). Consequently, bands exhibiting a high absolute load value are selected, as they strongly correlate with the principal components and represent the most informative characteristics of the data.

#### 2.3.2 Band selection based on ACO

Through the steps of initializing parameters, designing a fitness function, constructing paths, and updating pheromones, the ACO gradually optimizes a subset of wavelengths to enhance the predictive performance of the model (35). During the iterative process, ants select wavelength variables based on pheromone concentration and heuristic information along the path. The pheromone concentration highlights high-quality paths through an update mechanism (36), which guides the ants in identifying key wavelengths and excluding redundant variables. The fitness function is typically defined as a PLSR model (37), which evaluates prediction accuracy using metrics such as mean square error and coefficient of determination. Ultimately, the algorithm mines optimal wavelength combinations through a combination of global search and local optimization, thereby enhancing the model's predictive ability and robustness. By adjusting parameters such as the number of ants and the pheromone volatility coefficient, the ACO can effectively balance exploratory and convergence speeds (38), making it applicable to the challenge of selecting complex, high-dimensional wavelength data.

In the context of band selection using ACO, each ant progressively selects a series of bands to form a combination, which constitutes the ant's path. This path signifies the journey of the ant from the starting point to a complete band combination, achieved through the selection of various bands. If a particular path is frequently traversed by previous ants and demonstrates superior performance, the pheromone concentration along this path will increase, thereby attracting more ants to favor this route. Conversely, heuristic information reflects the relationship or superiority between the current band and the subsequent band, such as the standard deviation of the band. Each ant's path is evaluated based on its performance, and pheromone concentration on paths that yield favorable results will rise, further encouraging other ants to select these paths.

#### 2.3.3 Band selection based on FOACO

In traditional calculus, the most commonly utilized operations are integro-order differentiation and integration, including first-order and second-order derivatives (18). These operations describe the rate of change of a function at a specific point; for instance, the first-order derivative indicates the rate of change, while the second-order derivative reflects the curvature of the curve, signifying acceleration or curvature. For example, for the function *y* = *f*(*x*)and its first order derivative$y^{\prime }=f^{\prime }(x)=\lim_{\Delta x\to 0}\frac{f(x+\Delta x)-f(x)}{\Delta \;x}$.

Fractional calculus is an extension of traditional integer calculus, focusing on the quantitative analysis of integration and differentiation of functions with non-integer orders, which can be real numbers, complex numbers, or even functions of variables (39). For instance, Dxαaf(x)represents the *a* derivative of *f*(*x*), where *a* and *x* denote the upper and lower bounds of the integral (or derivative), and α signifies the fractional order. When *a* is an integer, fractional calculus reduces to the familiar integer calculus; specifically, when α = 2, Dxαaf(x) corresponds to the second derivative of the function. The exploration of fractional calculus began in 1695 when the German mathematician Leibniz and the French mathematician Leibniz discussed the implications of a derivative of order 1/2 (40).

**Figure 2:**
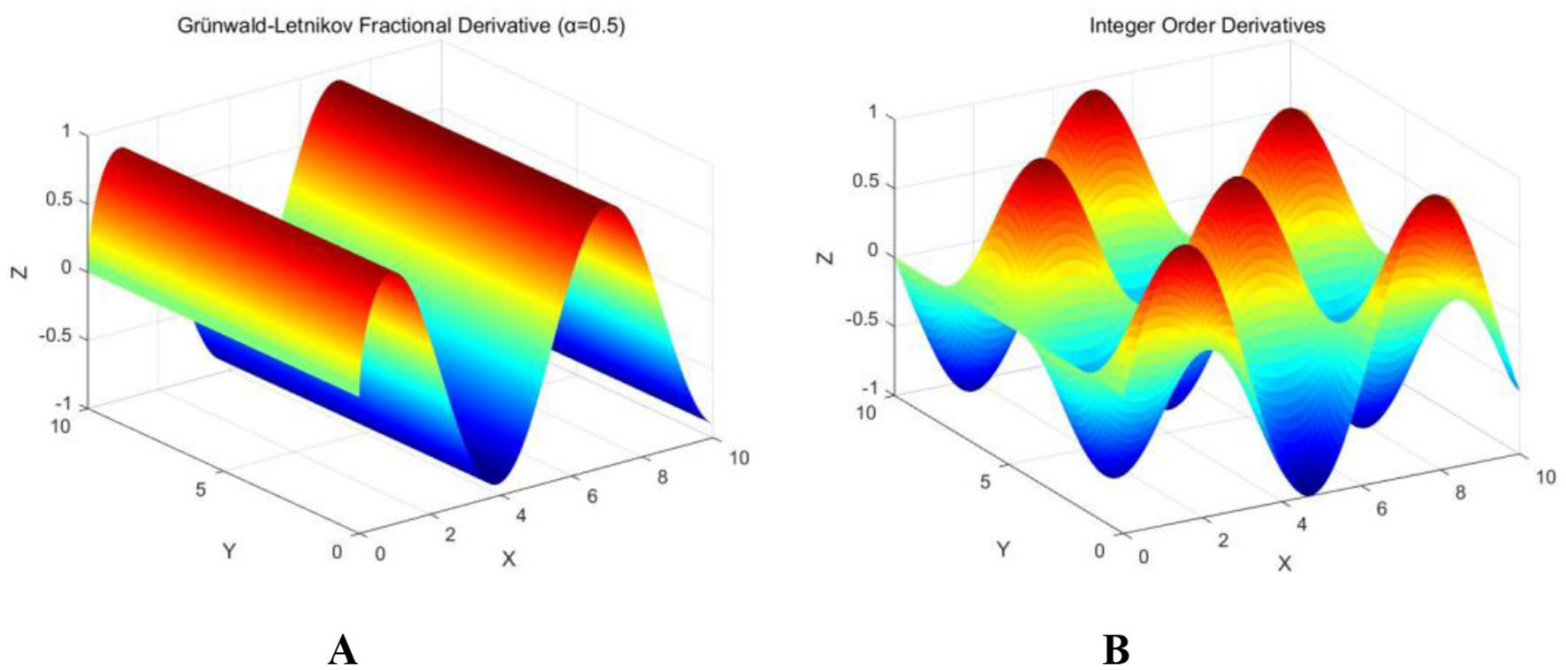
Integral three-dimensional images. **(A)** Fractional calculus; **(B)** Integer Order Calculus.

Figure 2A illustrates that the fluctuations generated by fractional calculus exhibit significant smoothness, with a more uniform and stable color gradient, minimal oscillation, and an absence of sudden fluctuations. This gradual change is softer compared to the traditional integer-order derivative in both temporal and spatial contexts, which aids in progressively mitigating the influence of local extrema, thereby reducing the likelihood of falling into local optima and enhancing the algorithm's global search capability. In contrast, the integer-order derivative depicted in Figure 2B demonstrates sharp and direct fluctuations, steep color gradients, high oscillation frequencies, and pronounced multi-peak and multi-valley characteristics. While this behavior is more suitable for sensitive analyses of instantaneous changes and local phenomena, it also renders the system more susceptible to noise and short-term fluctuations, resulting in unstable outcomes.

The FOACO is an optimization method derived from the classical ACO, designed to enhance search efficiency and stability in high-dimensional feature selection tasks. In the ACO, ants select paths based on the pheromone concentration of the current route and a heuristic function. Pheromone updates typically rely on an accumulation mechanism of integer order (21). However, this mechanism can lead to excessive sensitivity or instability in pheromone updates, causing the algorithm to become trapped in local optima or exhibit slow convergence. Common definitions of fractional calculus include the Grünwald-Letnikov (18), Riemann-Liouville (41), and Caputo (42) definitions. In this study, we employed the Grünwald-Letnikov definition of fractional calculus to update the pheromone concentration within the algorithm. Grunwald-Letnikov defines the formula as follows:


(2) $$\begin{array}{l} \begin{array}{c} D^{\alpha }f\left(t\right)=\lim_{h\;\to \;0}\frac{1}{h^{\alpha }}\;\overset{\left\lfloor t/h\right\rfloor }{\underset{k\;=\;0}{\sum }}{\left(-1\right)}^{k}\left(\frac{\alpha }{k}\right)\;f\left(t\;-\;kh\right) \end{array} \end{array}$$

In Equation 2, *D*^α^*f*(*t*) represents the fractional derivative of the function *f*(*t*), which is defined at time *t*. *D*^α^ denotes the fractional derivative operator, where α (0 < α ≤ 1) is a real number, and indicates the order of the fractional derivative. Where *h* is the discrete time step, and the limit *h* → 0 ensures that the discrete approximation can fully approximate the continuous fractional derivative; The fractional binomial coefficient $\left(\frac{\alpha }{k}\right)$ is defined as $\left(\frac{\alpha \left(\alpha -1\right)\cdots \left(\alpha -k+1\right)}{k!}\right)$. Under non-integer order α, reasonable weights are assigned to historical function values, reflecting the influence of system memory effect. The alternating sign of (−1)^*k*^ in the summation term helps to balance the positive and negative contributions, so that the summation process can accurately simulate the behavior of traditional differential operations. This formula not only expands the theoretical scope of classical calculus, but also provides a solid theoretical foundation for fractional-order ant colony algorithms to deal with complex and dynamically changing system problems in the field of optimization and control.

The Grünwald-Letnikov fractional derivative enhances the historical behavior of the modeling function by incorporating a non-integer order smoothing factor through weighted summation. By leveraging the smoothness of the Grünwald-Letnikov fractional derivative, the ants' path selection is influenced not only by the current pheromone concentration but also by the smoothed historical trend of pheromone changes. This approach facilitates a more time-dependent and stable decision-making strategy. Following the introduction of the fractional order pheromone decay mechanism and the fractional derivative, the revised pheromone update formula is presented as follows:


(3) $$\tau_{ij}(t\;+\;1)=(1\;-\;\rho )\cdot \tau_{ij}(t)\;+\;\sum_{t^{\prime }=\;0}^{t}\left(\frac{\alpha }{t^{\prime }}\right){\left(-1\right)}^{t^{\prime }}\cdot \tau_{ij}(t\;-\;t^{\prime })$$

In Equation 3, ρ denotes the pheromone evaporation coefficient, which quantifies the natural attenuation of pheromone. The binomial coefficient $\left(\frac{\alpha }{t^{'}}\right)$ assigns a weight to a historical time point, signifying that the current time point is influenced by the values of preceding time points. $\tau_{ij}(t-t^{'})$ represents the pheromone concentration at various time steps in the history, specifically from node *i* to node *j*.

After the pheromone is updated by the fractional derivative, the transition probability of the ants is adjusted accordingly, and the transition probability function is modified to the fractional form:


(4) $$\begin{array}{l} \begin{array}{c} p_{ij}\left(t\right)=\frac{{\left(D^{\alpha }\;\tau_{ij}\;\left(t\right)\right)}^{\alpha }\cdot \;\eta_{ij}^{\beta }}{\underset{k\;\in \;allowed}{\sum }\;{\left(D^{\alpha }\;\tau_{ik}\;\left(t\right)\right)}^{\alpha }\cdot \;\eta_{ik}^{\beta }}\\ \; \end{array} \end{array}$$

In Equation 4, *D*^α^ represents the fractional differential operator, indicating that the fractional derivative is applied to the pheromone concentration τ_*ij*_. τ_*ij*_ denotes the pheromone strength on the path (*i, j*), while η_*ij*_ refers to the heuristic information for the path (*i, j*), which is typically the inverse of the distance. Parameters α and β regulate the significance of the pheromone and the heuristic information, respectively.

In this manner, ants can make more coherent and globally optimized decisions by utilizing the history of pheromone changes. This approach effectively balances the influence of local and global information, allowing ants to consider both past experiences and global data when selecting a path. Consequently, this enhances the global search capability of the algorithm, helps to avoid premature convergence, and facilitates the identification of a more optimal path.

Based on the aforementioned theoretical framework, the overall flow of the FOACO wavelength selection algorithm is constructed as follows:

(1) Initialization of Parameters: Parameters such as the number of ants, the number of iterations, and the pheromone evaporation coefficient are initialized. Additionally, pheromone update parameters, including the weight of pheromone and heuristic information, are determined. The fractional order is set to manage the accumulation and decay of pheromone.
(2) Path Construction: Each ant begins from a randomly selected node (band), integrating pheromone concentration and heuristic information to progressively select bands and construct a path.
(3) Calculation of Fitness Value: The model computes the prediction error; a higher fitness value indicates a superior quality of band combination, which leads to more accurate prediction results.
(4) Pheromone Update: Pheromone concentration and path quality are used to update the pheromone levels, incorporating a fractional derivative to refine the control over pheromone accumulation and decay. Although pheromone values diminish over time, the update process is influenced by path fitness, with paths exhibiting higher fitness enhancing their pheromone concentration to attract more ants, thereby accelerating algorithm convergence and optimizing band selection.
(5) Calculation of Transition Probability: By combining pheromone concentration and heuristic information after fractional derivative processing, the selection probability for each band is calculated. This determines the subsequent band choice for each ant and facilitates the ongoing path construction. This step further refines the path selection process, aiding the ant colony in identifying the optimal band combination.

**Figure 3:**
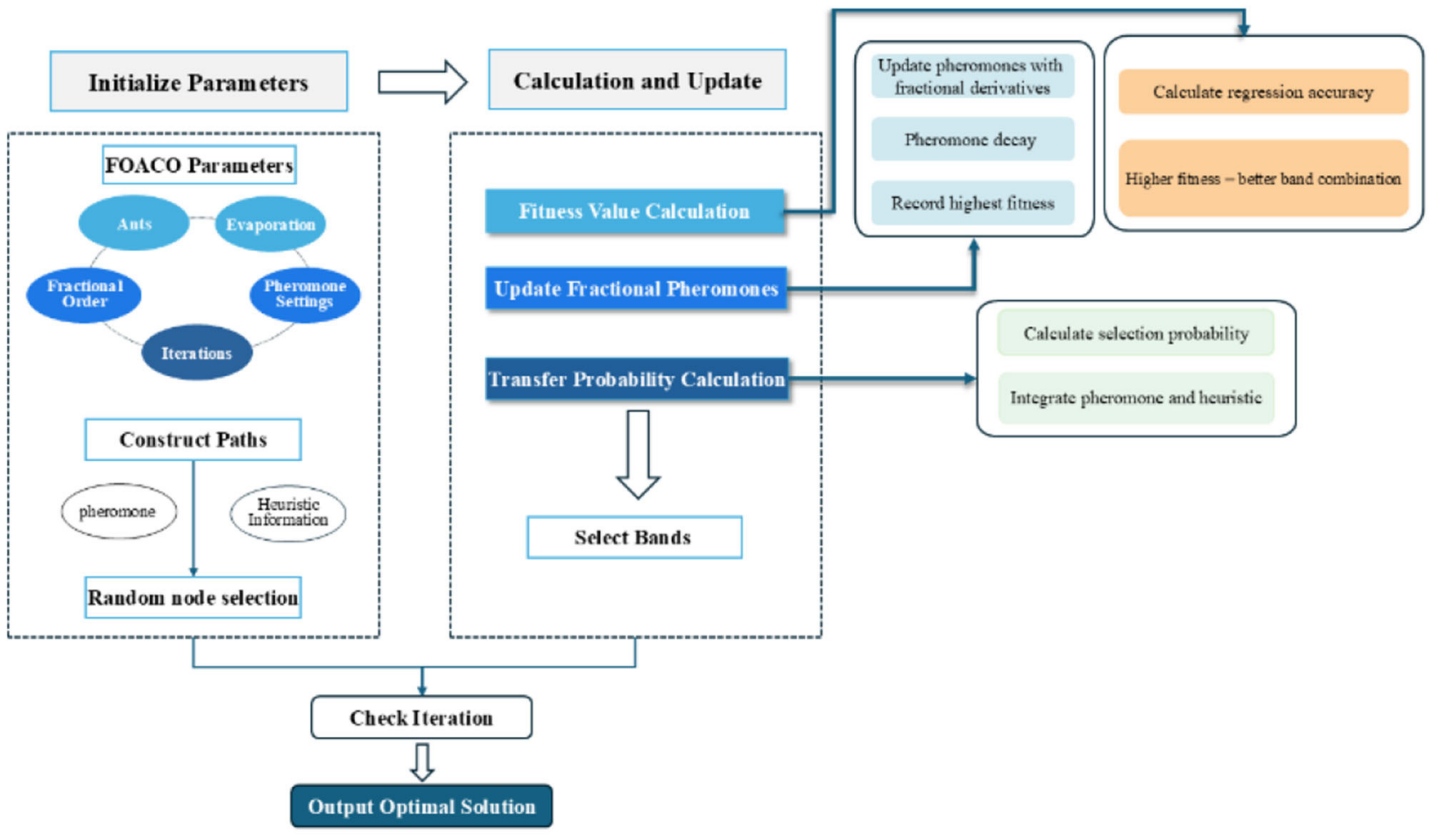
Flow model diagram for FOACO band selection.

Figure 3 shows the specific process of FOACO band selection.

### 2.4 Model evaluation methods

Model evaluation is a key part of the study, where the performance of the model is measured by a combination of multiple metrics for both the calibration and prediction sets. Specifically, the evaluation metrics include the coefficient of determination (*R*^2^), root mean square error (RMSE), relative prediction deviation (RPD), and mean absolute error (MAE), which are able to comprehensively assess the model's fitting effect, prediction accuracy and error level, and thus provide an in-depth comparison and analysis of the model's performance (43). In the calibration set (Cal) and prediction set (Pre) performance evaluation, the evaluation metrics of Cal are used to measure the fitting ability of the model on training data, while the evaluation metrics of Pre are used to reflect the prediction performance of the model on unknown data (44). In general, a better model should have high *R*^2^ and RPD on both calibration and prediction sets, as well as low values on RMSE and MAE, indicating that the model can not only fit the training data effectively, but also provide accurate prediction results.

## 3 Results and discussion

### 3.1 Selection of spectral representation and optimal preprocessing

**Figure 4:**
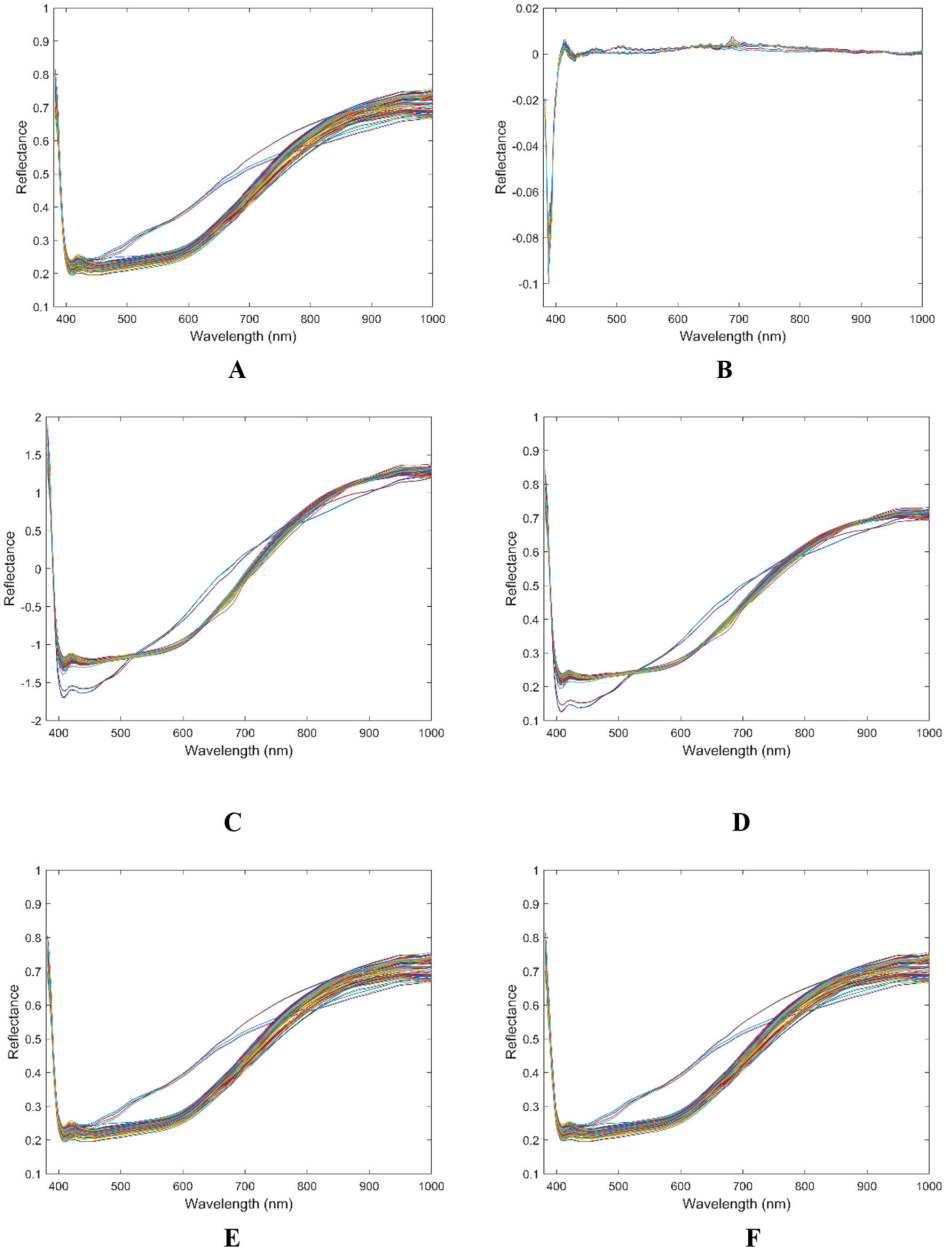
Flaxseed spectral reflectance curves. **(A)** Raw spectral curve of flaxseed; **(B)** 1stDer preprocess spectral curve. **(C)** SNV preprocess spectral; curve of flaxseed; **(D)** MSC preprocess spectral curve; **(E)** MA preprocess spectral curve of flaxseed; **(F)** SG preprocess spectral curve of flaxseed.

After measuring the protein content of 30 flaxseed varieties, the original spectral data, along with seven preprocessed datasets, were combined with the actual protein content data to develop a PLSR prediction model for flaxseed protein. The PLSR model enhances performance by calculating latent variables and selecting the optimal number of these variables through a cross-validation method. Subsequently, the cross-validation metrics, *R*^2^ and RMSE were employed to evaluate and determine the most effective preprocessing method. Table 3 presents the results, revealing that all preprocessing methods, with the exception of the first-order derivative, improve the model's accuracy compared to the original spectral modeling. Notably, the model demonstrates peak performance when the SG method is utilized as the preprocessing technique. For the calibration and validation sets, the *R*^2^ values are 0.9411 and 0.8794, respectively, while the RMSE values are 0.3456 and 0.5487, and the RPD values are 4.1188 and 2.8797. Consequently, SG has been identified as the most effective preprocessing method. Figure 4 illustrates the average spectra of the 30 different flaxseed varieties alongside the average spectrogram of five preprocessed samples. The SG method effectively smooths the spectral data and reduces noise, thereby enhancing the robustness and reliability of the data by minimizing the influence of outliers, which in turn improves the model's stability and accuracy. Therefore, the SG preprocessing method is employed for further feature extraction.

**Table 3:** Prediction results of partial least squares regression model based on raw and preprocessed spectra.

**Preprocessing methods**	**Cal**				**Pre**			
	***R* ^2^**	**RMSE**	**RPD**	**MAE**	***R* ^2^**	**RMSE**	**RPD**	**MAE**
None	0.9436	0.3380	4.2114	0.2735	0.8026	0.7296	2.1658	0.6005
1st	0.9107	0.4254	3.3459	0.3499	0.7257	0.8275	1.9095	0.6361
SNV	0.9095	0.4281	3.3248	0.3545	0.8303	0.6510	2.4274	0.5033
MSC	0.9096	0.4278	3.3267	0.3603	0.8394	0.6333	2.4952	0.4912
MA	0.9283	0.3810	3.7354	0.3221	0.8535	0.6047	2.6129	0.4809
SG	0.9411	0.3456	4.1188	0.2834	0.8794	0.5487	2.8797	0.4918

R^2^, efficient of determination; RMSE, Root Mean square error; RPD, relative forecast deviation; MAE, Mean Absolute error.

### 3.2 Model and performance analysis

#### 3.2.1 Selection of characteristic wavelength

**Figure 5:**
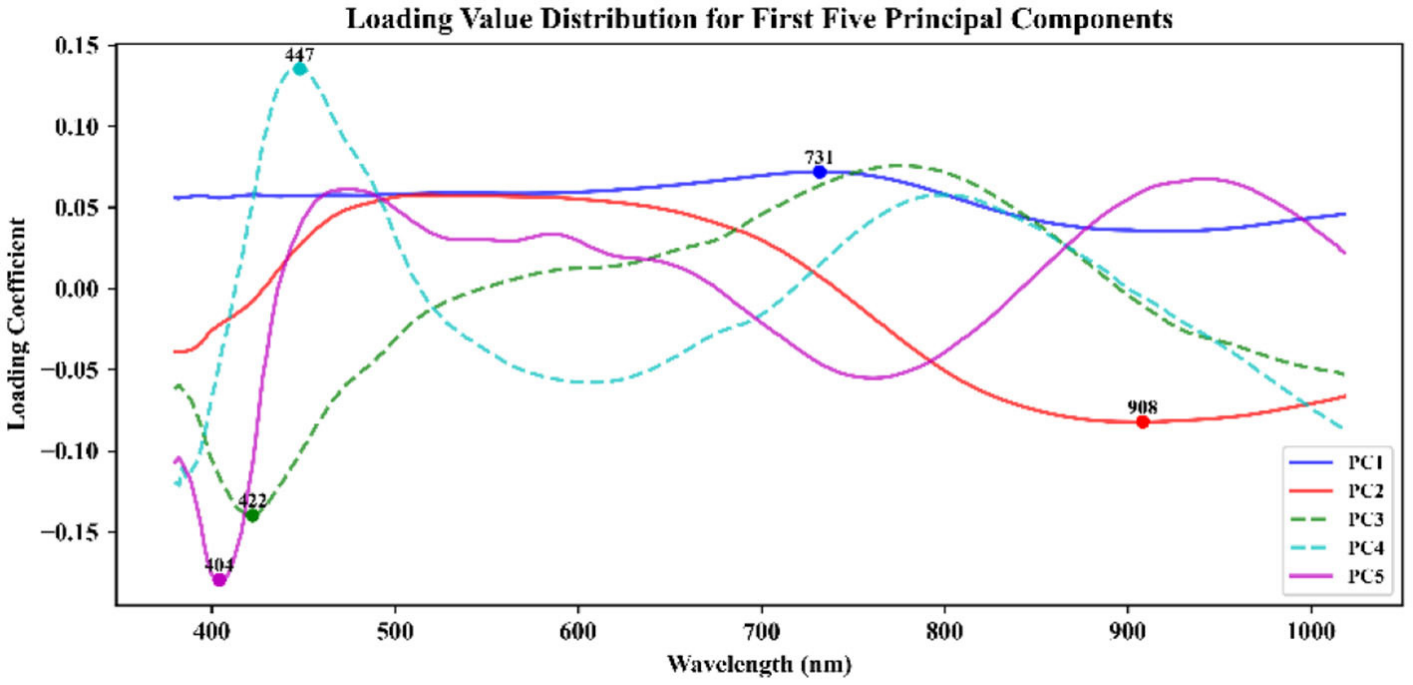
Characteristic wavelength extraction based on PCA-loading.

**Figure 6:**
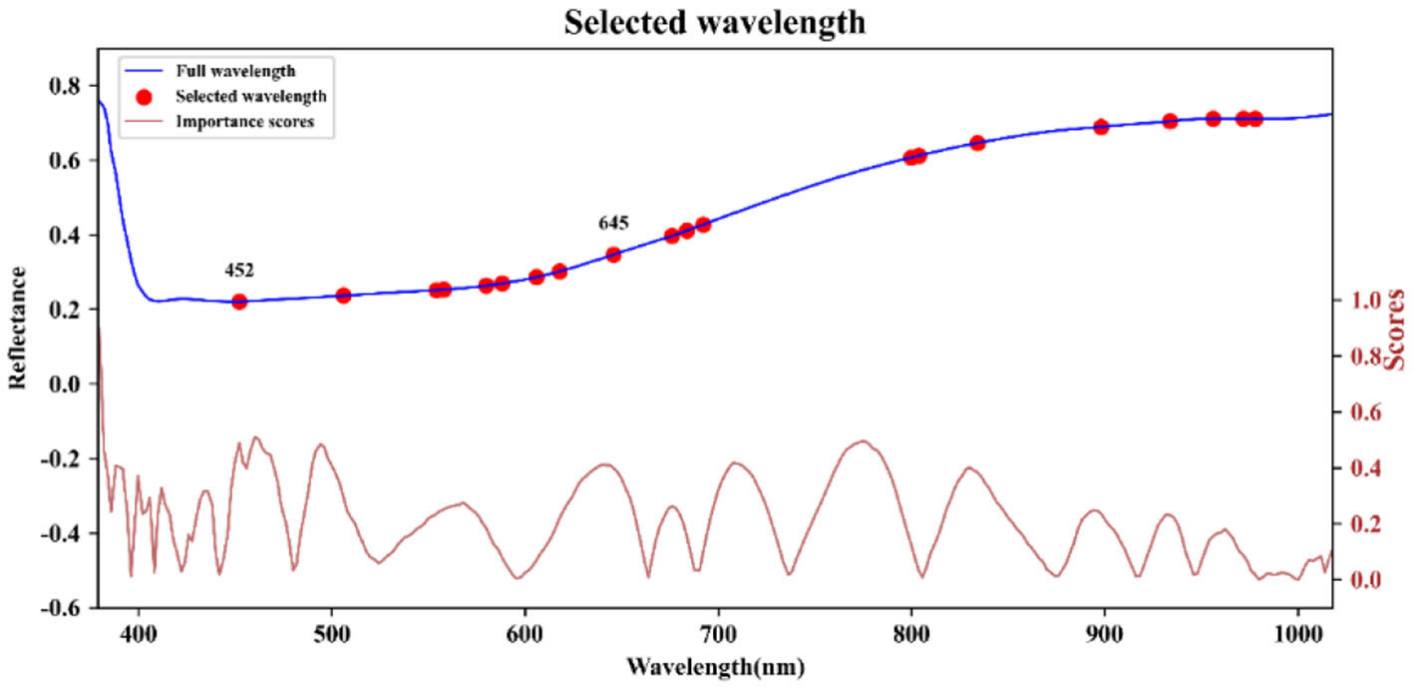
Key bands based on ACO selection.

**Figure 7:**
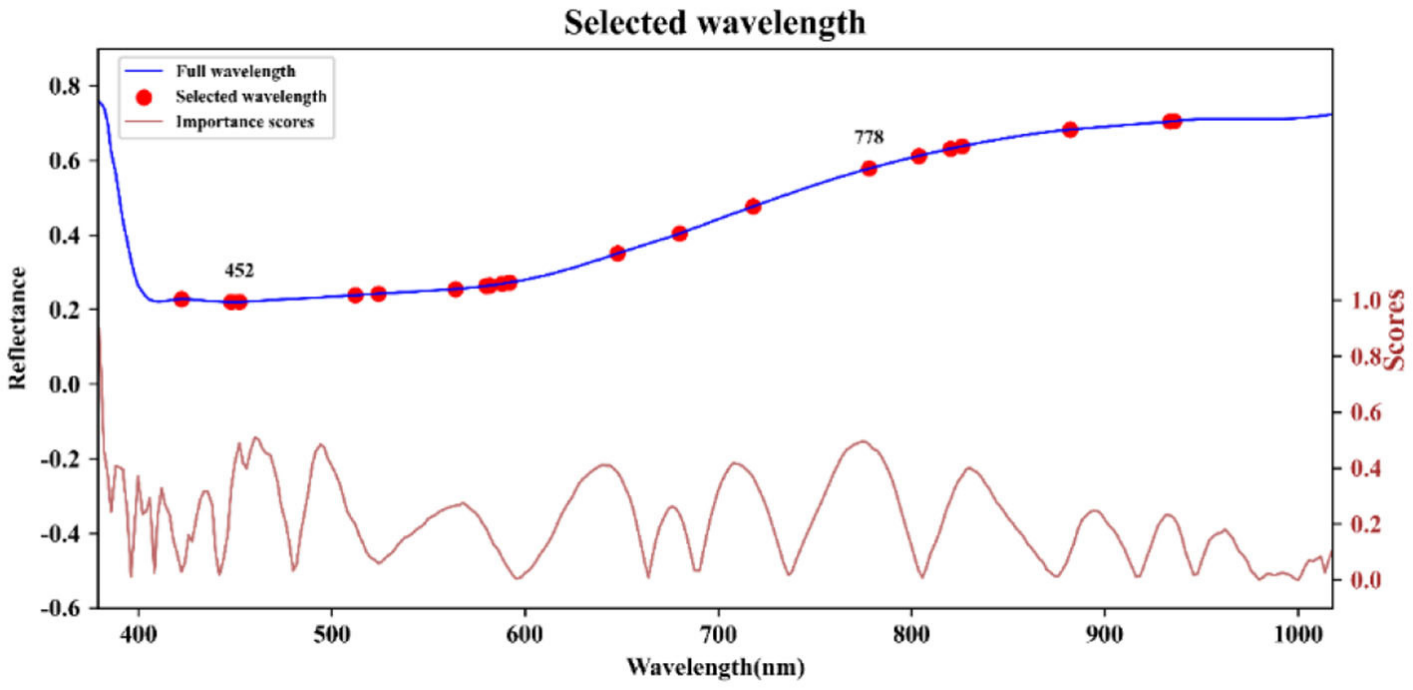
Key bands based on FOACO selection.

After conducting PCA on the sample spectra of the training set, the principal component with the highest cumulative contribution rate among the first five components is selected. Subsequently, the characteristic wavelength exhibiting the highest correlation with the corresponding principal components is identified based on the load values of these components. The distribution of feature wavelengths selected using the PCA-loading method across the full spectrum is illustrated in Figure 5. ACO gradually selects bands to maximize classification performance or minimize redundancy by simulating ant foraging behavior and utilizing pheromone concentration alongside heuristic information, such as band relevance and redundancy. During the band selection process, each ant is guided by the pheromone concentration, favoring bands with stronger pheromone signals, thus progressively moving toward the optimal solution. The key bands identified through the ACO method are illustrated in Figure 6. FOACO enhances the ACO by incorporating fractional derivatives to optimize the pheromone concentration update method, resulting in improved global exploration capabilities and a smoother search path for band selection. During the search process, ants utilize both pheromone concentration and heuristic information, gradually converging on an optimal band combination characterized by high correlation and low redundancy. The key bands selected through the FOACO method are illustrated in Figure 7. In this study, both ACO and FOACO are configured to select 20 optimal bands. Subsequently, the data from these optimized bands are input into regression models, and the performance of various band selection methods is systematically evaluated using the PLSR and MLR models.

#### 3.2.2 Comparison of models

PLSR and MLR models are employed to model and analyze the selection results of various band selection methods, including PCA, ACO, and FOACO, while comparing the predictive performance of each method. As indicated in Table 4, the PLSR model demonstrates that the FOACO method yields the best results, achieving a prediction set *R*^2^ of 0.9205, an RPD of 3.2582, an RMSE of 0.4468, and an MAE of 0.4226. When compared to the original data without selected bands, *R*^2^ increases by 14.8%, RPD improves by 50.4%, RMSE decreases by 38.4%, and MAE decreases by 29.64%, highlighting the significant impact of FOACO on enhancing the prediction performance of the PLSR model. In the MLR model, FOACO also exhibits strong performance, with a prediction set *R*^2^ of 0.9248, an RPD of 3.6458, an RMSE of 0.4346, and an MAE of 0.3259. Relative to the FOACO-PLSR, *R*^2^ increases by ~0.47%, RMSE decreases by around 2.73%, RPD improves by about 11.91%, and MAE decreases by ~22.86%. These findings indicate that the FOACO-MLR model outperforms the combined PLSR model across all evaluation metrics, particularly in terms of prediction performance. The analysis suggests that the FOACO method presents significant advantages in both modeling approaches, effectively enhancing the accuracy and stability of spectral data modeling, thereby providing more reliable prediction outcomes. While the ACO and PCA methods contribute to some extent in improving model performance, the FOACO method demonstrates superior efficacy in enhancing both the accuracy and stability of the model.

**Table 4:** Prediction results [values of *R*^2^, RMSEP, relative prediction deviation (RPD), mean absolute error (MAE)] using partial least squares regression (PLSR) and multiple linear regression (MLR) models at full wavelength and selected wavelengths for principal component analysis (PCA), ant colony optimization (ACO), and fractional order ant colony optimization (FOACO).

	**Wavelength selection**	**Calibration**				**Prediction**			
**Models**		***R* ^2^**	**RMSE**	**RPD**	**MAE**	***R* ^2^**	**RMSE**	**RPD**	**MAE**
PLSR	None	0.9436	0.3380	4.2114	0.2735	0.8026	0.7296	2.1658	0.6005
	PCA	0.9209	0.4060	3.7833	0.3025	0.8404	0.5337	3.1826	0.4979
	ACO	0.9277	0.3755	3.8243	0.2697	0.8958	0.5115	3.2128	0.4063
	FOACO	0.9306	0.3677	4.0059	0.2805	0.9205	0.4468	3.2582	0.4226
MLR	None	^*^	^*^	^*^	^*^	^*^	^*^	^*^	^*^
	PCA	0.9326	0.3626	0.2870	3.8607	0.8808	0.5471	3.1200	0.3912
	ACO	0.9351	0.3558	3.9246	0.2853	0.9124	0.4690	3.3787	0.3569
	**FOACO**	**0.9377**	**0.3485**	**4.1231**	**0.2678**	**0.9248**	**0.4346**	**3.6458**	**0.3259**

The asterisk symbol (^*^*) denotes exclusion of 320-band data from MLR modeling due to the method's requirement that predictor variables must be fewer than available samples. Bold values highlight optimal performance metrics across comparative methods.

**Figure 8:**
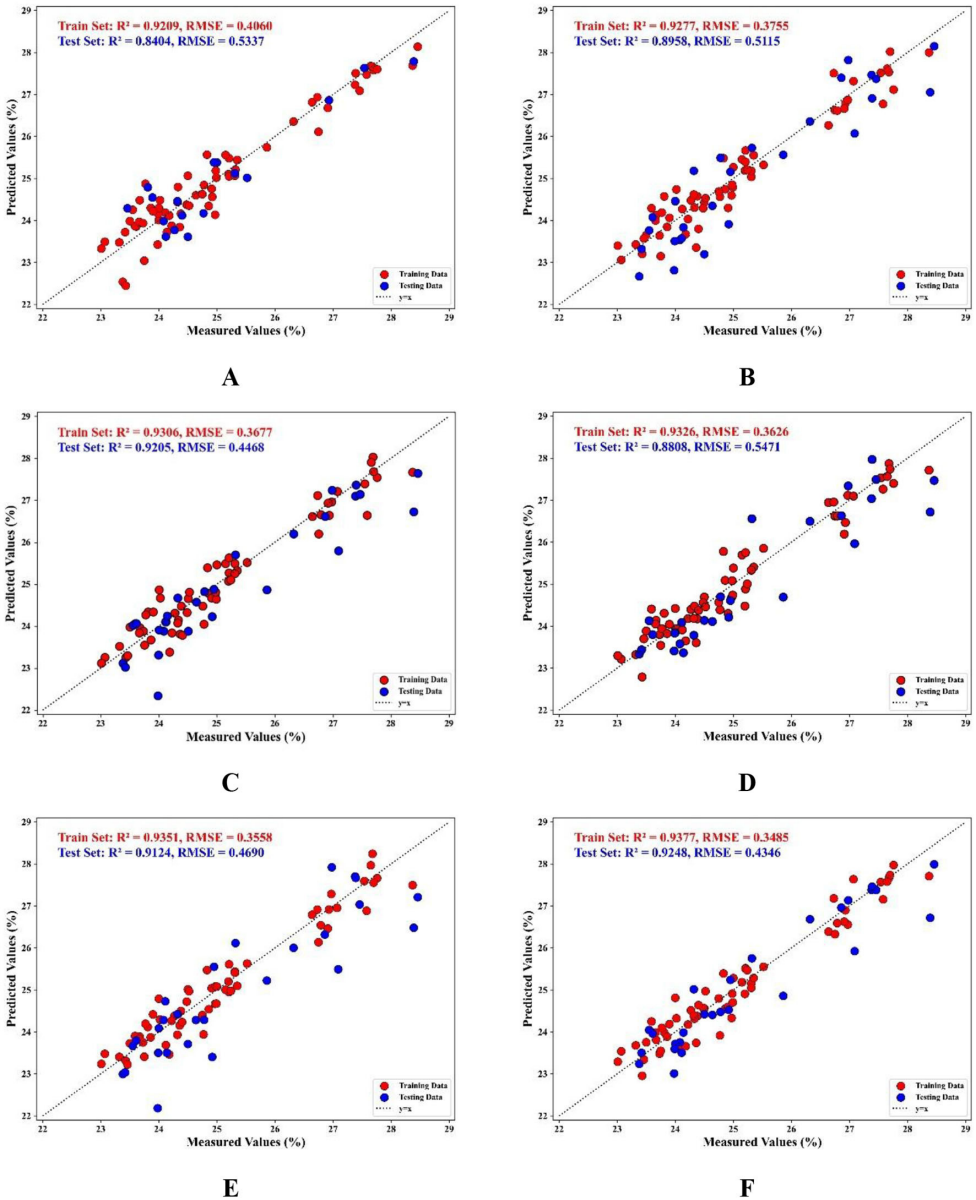
PLSR and MLR prediction results based on selected wavelengths; **(A)** the effect of PLSR prediction model based on PCA selected bands; **(B)** the effect of PLSR prediction model based on ACO selected bands; **(C)** the effect of PLSR prediction model based on FOACO selected bands; **(D)** the effect of MLR prediction model based on PCA selected bands; **(E)** the effect of MLR prediction model based on ACO effects of MLR prediction models for selected bands; **(F)** effects of MLR prediction models for selected bands based on FOACO.

To visualize the improvement in the predictive performance of the model, a scatter plot is employed to illustrate the degree of fit between the predicted values and the true values. The scatter plot effectively highlights the strengths and weaknesses of each method by comparing the fit of the predicted values to the true values. A distribution of points that is closer to the 45° diagonal indicates better predictive performance of the model (45). By observing Figure 8, which depicts the correlation between predicted and true values for each model, it is evident that the FOACO method demonstrates a better fit in the MLR model compared to PCA and ACO. The proximity of the predicted values to the true values is significantly higher, indicating a superior fit.

#### 3.2.3 Model diagnostics

**Figure 9:**
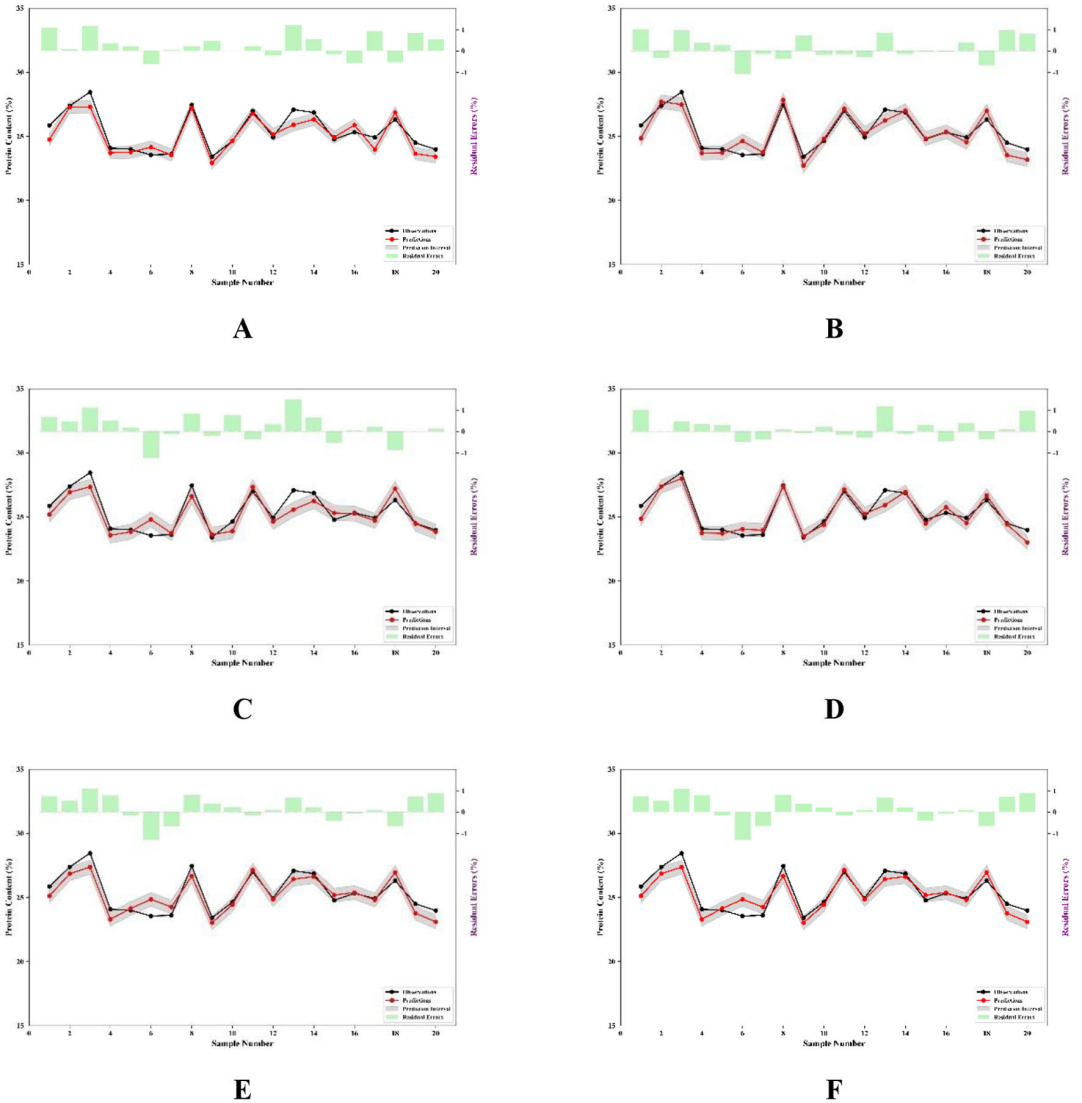
Prediction intervals and residuals based on selected wavelengths. **(A)** PLSR prediction intervals and residuals of selected bands based on PCA; **(B)** PLSR prediction intervals and residuals based on ACO selected bands; **(C)** PLSR prediction intervals and residuals of the selected bands based on FOACO; **(D)** The MLR prediction intervals and residuals of the selected bands based on PCA; **(E)** MLR prediction intervals and residuals based on ACO selected bands; **(F)** MLR prediction intervals and residuals of bands selected based on FOACO.

The use of residual plots enhances the credibility of model results. These plots not only provide a visual representation of the model's predictive performance but also assist in identifying error distributions, quantifying uncertainty, and optimizing model performance (46). In this study, the FOACO method demonstrated excellent performance in both PLSR and MLR models. As shown in Figures 9A–E, a systematic comparison of prediction intervals and residuals highlights significant differences among the PCA-based, ACO-based, and FOACO-based approaches. Specifically, the PLSR and MLR models using PCA (Figures 9A, D) exhibit broader prediction intervals and greater residual dispersion, indicating limitations in conventional dimensionality reduction. In contrast, ACO (Figures 9B, E) reduces residual magnitudes compared to PCA, though residual variability remains. Building on the improvements observed in the FOACO-PLSR model (Figure 9C), where residuals cluster near zero and prediction intervals narrow, the FOACO-MLR model (Figure 9F) achieves the most significant error control, demonstrating the smallest residual magnitudes and the tightest prediction intervals among all comparative methods. The comparison of residual plots suggests that FOACO employs a more efficient optimization mechanism, substantially enhancing model performance-particularly in improving accuracy and controlling prediction errors. These results further illustrate that the FOACO method not only improves prediction accuracy but also effectively reduces model uncertainty, thereby providing more reliable and stable prediction outcomes for spectral data modeling.

## 4 Conclusion

This study verified the feasibility of vision-near-infrared hyperspectral imaging in the determination of protein content in 30 flax seed varieties. A novel, simplified and stable protein content evaluation model was constructed by selecting characteristic wavelength with different algorithms. In the process of characteristic wavelength selection, compared with traditional PCA and classical ACO methods, FOACO method shows obvious advantages in data dimensionality reduction, error control and model stability under both PLSR and MLR models. In particular, the combination of FOACO and MLR is superior in terms of overall prediction accuracy, error control and model robustness. The results showed that wavelength selection based on hyperspectral imaging technology combined with FOACO method and prediction model constructed by MLR model effectively simplified the spectral data dimension and improved the prediction ability of the model, providing a new technical idea for rapid and non-destructive detection of flax seed protein content. It also provides strong support for the quality testing and food safety management of other agricultural products.

## Data Availability

The raw data supporting the conclusions of this article will be made available by the authors, without undue reservation.
